# High-intensity interval training and hyperoxia during chemotherapy: A case report about the feasibility, safety and physical functioning in a colorectal cancer patient

**DOI:** 10.1097/MD.0000000000011068

**Published:** 2018-06-15

**Authors:** Nils Freitag, Pia Deborah Weber, Tanja Christiane Sanders, Holger Schulz, Wilhelm Bloch, Moritz Schumann

**Affiliations:** a Clinical Exercise Science, Department of Sport and Health Sciences, University of Potsdam; b Department of Molecular and Cellular Sport Medicine; c Department of Preventive and Rehabilitative Sport Medicine, Institute of Cardiovascular Research and Sport Medicine, German Sport University Cologne; d Clinical Centre for Oncological and Hematological Medicine Frechen, Germany.

**Keywords:** carcinoma, chemo-toxicity, exercise therapy, fatigue, gastrointestinal cancer, heart rate variability, high-intensity interval training, solid tumor

## Abstract

**Introduction:**

We conducted a case study to examine the feasibility and safety of high-intensity interval training (HIIT) with increased inspired oxygen content in a colon cancer patient undergoing chemotherapy. A secondary purpose was to investigate the effects of such training regimen on physical functioning.

**Case presentation:**

A female patient (51 years; 49.1 kg; 1.65 m; tumor stage: pT3, pN2a (5/29), pM1a (HEP), L0, V0, R0) performed 8 sessions of HIIT (5 × 3 minutes at 90% of W_max_, separated by 2 minutes at 45% W_max_) with an increased inspired oxygen fraction of 30%. Patient safety, training adherence, cardiorespiratory fitness (peak oxygen uptake and maximal power output during an incremental cycle ergometer test), autonomous nervous function (i.e., heart rate variability during an orthostatic test) as well as questionnaire-assessed quality of life (EORTC QLQ-C30) were evaluated before and after the intervention.

No adverse events were reported throughout the training intervention and a 3 months follow-up. While the patient attended all sessions, adherence to total training time was only 51% (102 of 200 minutes; mean training time per session 12:44 min:sec). VO_2peak_ and W_max_ increased by 13% (from 23.0 to 26.1 mL min kg^−1^) and 21% (from 83 to 100 W), respectively. Heart rate variability represented by the root mean squares of successive differences both in supine and upright positions were increased after the training by 143 and 100%, respectively. The EORTC QLQ-C30 score for physical functioning (7.5%) as well as the global health score (10.7%) improved, while social function decreased (17%).

**Conclusions:**

Our results show that a already short period of HIIT with concomitant hyperoxia was safe and feasible for a patient undergoing chemotherapy for colon cancer. Furthermore, the low overall training adherence of only 51% and an overall low training time per session (∼13 minutes) was sufficient to induce clinically meaningful improvements in physical functioning. However, this case also underlines that intensity and/or length of the HIIT-bouts might need further adjustments to increase training compliance.

## 1 Introduction

Chemotherapy is a well-advised, first-line drug treatment for colorectal cancer (CRC) and is considered essential for a progression-free and overall survival in CRC patients.^[1]^ However, chemotherapy is also well known for its strong short- and long-term side-effects,^[2]^ such as chemo-toxicity (e.g., nausea or leukopenia), cognitive impairments and cancer-related fatigue.^[2,3]^ The consequences of these treatment-induced side-effects are a sedentary lifestyle and decreased performance capacity, ultimately leading to higher rates of depression, a lower quality of life (QoL) and increased overall mortality.^[4]^ Physical inactivity and psychosocial problems may also negatively influence the ability to cope with activities of daily living and impair therapy compliance.^[4]^

Physical exercise is well known to potentially reduce the chemotherapy-induced side effects in CRC, and may improve physical functioning and well-being.^[5,6]^ However, the majority of studies investigating the effects of aerobic exercise on cancer-related outcomes have applied only low to moderate exercise intensities.^[7]^ This is somewhat surprising because high-intensity interval training (HIIT) has previously been shown to induce superior cardiovascular benefits by increasing cardiorespiratory fitness and reducing risk factors compared to low-intensity training not only in healthy but also in diseased populations, such as chronic heart failure,^[8]^ type 2 diabetes,^[9]^ or multiple sclerosis.^[10]^ While the number of studies investigating HIIT in cancer, such as testicular cancer,^[11]^ and CRC,^[12]^ is increasing, the majority of these studies have included cancer survivors who are no longer on treatment. The reasons for a lack of data dealing with HIIT during cancer treatment might be related to the chemotherapy-induced fatigue and concomitant perceived exertion even during low-intensity training. To counteract this, increasing the inspired fraction of oxygen (FiO_2_, hyperoxia) may reduce the subjective perceived exertion during exercise and has been shown to enhance performance and recovery both in healthy^[13]^ and diseased populations.^[14–16]^ In particular, exercise in hyperoxia has been shown to reduce blood lactate concentrations, ventilation rates, fatigue and cardiac output, while at the same time increasing arterial oxygen saturation and improving aerobic metabolism in patients with chronic obstructive pulmonary disease,^[14]^ chronic heart failure^[16]^ and type 2 diabetes.^[15]^ However, to the best of our knowledge this has not yet been investigated in cancer patients.

Reduced breathlessness and blood lactate concentrations may, in turn, enhance training adherence and the willingness to withstand higher intensities over a prolonged time.^[15,17]^ Thus, it may be hypothesized that increased FiO_2_ might enhance therapy compliance in cancer patients undergoing chemotherapy, which would allow to incorporating higher exercise intensities. Hence, the purpose of this case report was to examine the feasibility, safety and adaptations in physical function of HIIT combined with hyperoxia in a CRC patient, providing a novel therapy approach in cancer patients undergoing chemotherapy.

## 2 Case study

The patient was a 52-year-old Caucasian inactive woman with a stenosed right sided, synchronously hepatic metastatic colon carcinoma (tumor stage: pT3, pN2a (5/29), pM1a (HEP), L0, V0, R0). The diagnosis of colorectal cancer was given in June 2017. The patient received chemotherapy (FOLFOX4 [oxaliplatin, folinic acid, and infusional 5-fluorouracil]) as a first-line treatment after the surgery. Furthermore, the patient presented cardiovascular risk factors, such as arterial hypertension (I10.90), hyperlipidemia (E78.2) and daily consumption of alcohol and nicotine. Arterial hypertension and hyperlipidemia were medically treated and controlled. No signs of neuropathy were present at the beginning of the study. The anthropometrics are presented in Table 1.

**Table 1:**
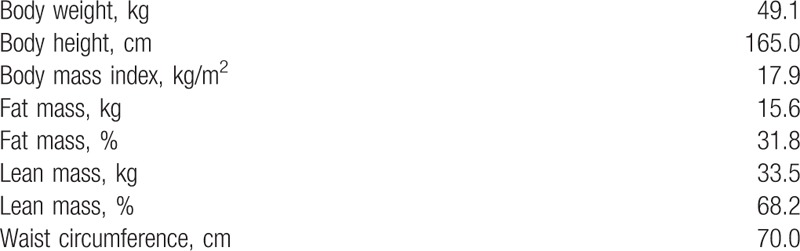
Patients body composition at baseline.

### 2.1 Investigations

The current study was carried out in accordance with the Declaration of Helsinki and received ethical approval by the Ethics Committee of the German Sport University, Cologne. The patient provided written informed consent prior to the first measurement and training. This case study is part of a larger trial, which is registered both at the German and the World Health Organization (WHO) trial registers (DRKS00011689) and accredited by the German Cancer Society (ST-U051). The present case study has been modified from a previous study design of our group published elsewhere.^[18]^

### 2.2 Training intervention

Eight HIIT sessions over a period of 4 weeks were carried out on a cycle ergometer (optibike 50 med, ergoline GmbH, Bitz, Germany). The training intensity was determined by a cardiopulmonary exercise test. Training consisted of 5 × 3 minutes high-intensity bouts at 90% of maximal power output (W_max_), separated by 2 minutes of active recovery at 45% of W_max_. Throughout each training session, the patient received additional oxygen (FiO_2_ 0.30). The gas–air mixture was directly supplied via a flexible tube from a compressor (HYPOXcontrol, Medicap homecare GmbH, Ulrichstein, Germany) to a full-face respiration mask, where it was mixed with ambient air to achieve the desired oxygen content.^[15]^ Training was synchronized with the individual drug administration and scheduled medical appointments. No training was conducted 24 hours following drug treatment. To allow for sufficient recovery, the exercise sessions were separated by at least 48 hours.

### 2.3 Measurements

#### 2.3.1 Outcome measurements

The primary outcomes of the present study were patient-related feasibility, safety (i.e., reported cardiac or oncological adverse events) as well as compliance to the chemotherapy and exercise training. Feasibility was assessed by trial completion, training adherence and compliance, program tolerance and patient safety. Training adherence and compliance were assessed using exercise diaries, indicating the number of training sessions performed and the total duration spent at high- and low training intensities. Program tolerance was assessed by the recordings of the rate of perceived exertion (RPE) during each training session.^[19]^ The safety of training was evaluated based on the reported numbers of adverse events during the intervention and follow-up.

Of secondary interest were changes in physical performance, body composition, lung function, basic blood count, autonomous nervous function and questionnaire-assessed fatigue and QoL.

#### 2.3.2 Cardiopulmonary exercise testing

Baseline measurements were performed before and after training. The cardiopulmonary exercise testing was conducted eight days past the chemotherapy administration. The patient performed a maximum stepwise incremental cardiopulmonary exercise test on a cycle ergometer (Excalibur sport, Lode BV, Groningen, the Netherlands) to determine the maximal aerobic capacity (VO_2peak_) and W_max_. In agreement with the guidelines provided by the WHO, the test started at an intensity of 25 W, which was then increased by 25 W every 2 minutes until voluntary exhaustion. The patient was requested to maintain a pedaling frequency of 65 revolutions per minute throughout the test. Electrocardiography was recorded constantly and was reviewed by a cardiologist. In addition, breathing gases were continuously recorded breath-by-breath using a gas exchange analyzer (ZAN600 CPET, nSpire Health GmbH, Oberthulba, Germany). Prior to the test, the device was calibrated for volume and fractional gas concentrations. In order to determine blood lactate concentrations, 20 μL of capillary blood samples were collected from the earlobe within the last 15 seconds at the end of each increment in order to assess blood lactate concentrations (Biosen S-Line, EKF Diagnostics, Barleben, Germany). Heart rate (HR) was measured using a commercially available wrist monitor and transmitter chest belt (Polar A300 Fitness/Activity Tracker, Polar Electro, Kempele, Finland). HR readouts were reported 10 seconds prior the end of the increment and directly after volitional termination of the test. Furthermore, the patient was asked to rate the subjective perceived exertion at the end of every increment on the Borg Scale. The patient was verbally encouraged to achieve maximal exhaustion and the test was stopped once the requested pedaling frequency was no longer maintained

#### 2.3.3 Body composition

Body composition (total body weight and fat- and lean mass, respectively) was assessed by bioelectrical impendence (BIA, Seca medical Body Composition Analyzer [mBCA 515], seca GmbH & Co.KG., Hamburg, Germany), using 2 electrodes on hands and feet, respectively. The body mass index (BMI) was calculated manually.

#### 2.3.4 Lung function

A spirometry (Easy on-Desk, ndd Medizintechnik AG, Zurich, Switzerland) was performed to determine the mechanical function of the lung and respiratory muscles by measuring the forced vital capacity (FVC) and the forced expiratory volume in the first second of exhalation (FEV_1_). Furthermore, the Tiffeneau–Pinelli index (FEV_1_/FVC) was calculated to assess the risk for obstructive and restrictive lung diseases.

#### 2.3.5 Basic blood count

Fasted venous blood samples were collected into EDTA-container (BD Vacutainer K2E) prior to the first and last basal measurement. A basic blood count was determined by fluorescent flow cytometry (Sysmex KX-21N, Sysmex Corporation, Kobe, Japan).

#### 2.3.6 Autonomous nervous function

Autonomous nervous function was assessed by heart rate variability (Firstbeat Technologies, Jvsäkylä, Finland) during an orthostatic test. The patient lay down in a supine position for 5 minutes and was requested to stand still in an upright position for a 3-min period afterwards. To assure artifact-free data, the heart rate variability (HRV) continuously analyzed excluding the first and last minute of measurement during supine and standing, respectively. The recorded HRV data were initially automatically corrected (Firstbeat Sports version 4.7.3.1) and, thereafter, manually checked by visual inspection. The following parameters were extracted from the recordings: resting HR during supine and standing, the beat to beat root mean square of successive R–R interval differences (RMSSD), spectral domain measures (low-frequency [LF] power (0.04–0.15 Hz), high-frequency (HF) power (0.15–0.4 Hz) and the LF/HF ratio.^[20]^

#### 2.3.7 Questionnaire-assessed patient-related outcomes

The following questionnaires were assessed before and after the intervention: QoL (European Organization for the Research and Treatment of Cancer Quality of Life Questionnaire [EORTC-QLQ-C30]), pain (Brief-Pain Inventory-Short Form [BPI-SF]), sleep quality (Pittsburgh Sleep Quality Index [PSQI]) and cognitive function (Functional Assessment of Cancer Therapy-Cognitive Function [FACT-Cog]). Additionally, perceived fatigue (Functional Assessment of Chronic Illness Therapy-Fatigue [FACIT-F]) was assessed before the first and the last HIIT session to gain information about the impact of the HIIT intervention on the subjective perception of fatigue.

#### 2.3.8 Habitual physical activity

Self-reported habitual physical activity was assessed via an activity diary. The patient was asked to report the daily mean time of physical activities of daily living (e.g., shopping, walking, riding a bicycle or duties at home like cleaning) on a weekly base.

### 2.4 Follow-Up

An additional follow-up of medical reports and adverse events was conducted 3 months past the postmeasurements of the intervention.

### 2.5 Data analysis

Primary and secondary outcomes are presented by absolute and relative changes from pre- to post-HIIT intervention. Data analyses were performed using Microsoft Excel 2011 (Microsoft, Redmond, WA).

### 2.6 Findings

#### 2.6.1 Feasibility and Safety

**Figure 1:**
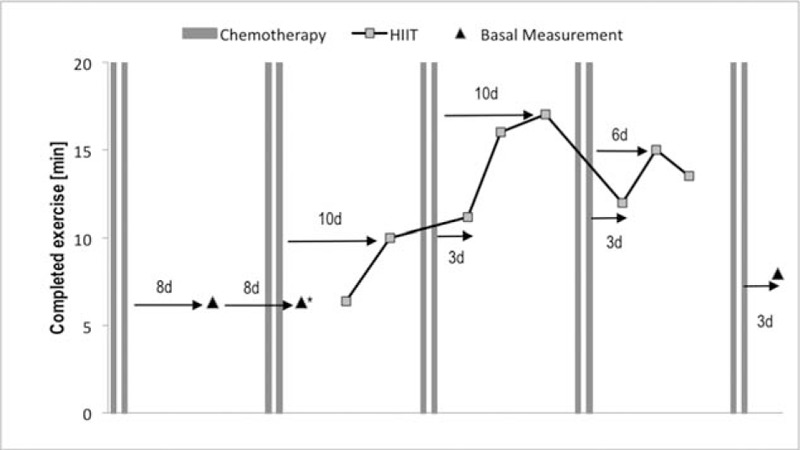
Timing of basal measurements, exercise interventions and drug administrations; Chemotherapy was administered on 2 consecutive days every week; d days ∗NOTE a second baseline incremental test was performed to clarify cardiovascular uncertainties indicated during the first CPET (the patient completed the same workload during the second baseline assessment). CPET = cardiopulmonary exercise testing.

The patient attended all 8 HIIT sessions and completed the training within 31 days (Fig. 1). On average, the patient was training every 3.9 ± 1.5 days (mean training time per session 12:44 min:sec out of 25 minutes).

The total training adherence was 51% (101:52 min:sec of 200 minutes). The total training time spent at high- and low-intensity bouts was 54% and 46% (65:02 min:sec of 120 min vs 36:50 min:sec of 80 minutes), respectively. Mean RPE values for each session are presented in Table 2.

**Table 2:**
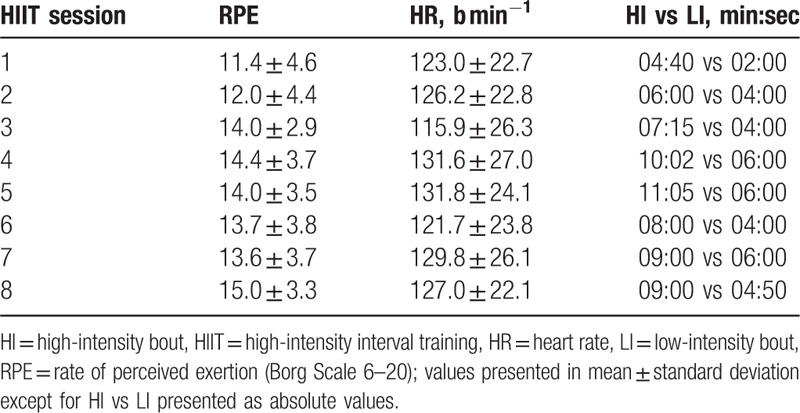
Program tolerance expressed by RPE, heart rate and total time spent at high- and low-intensity bouts.

**Figure 2:**
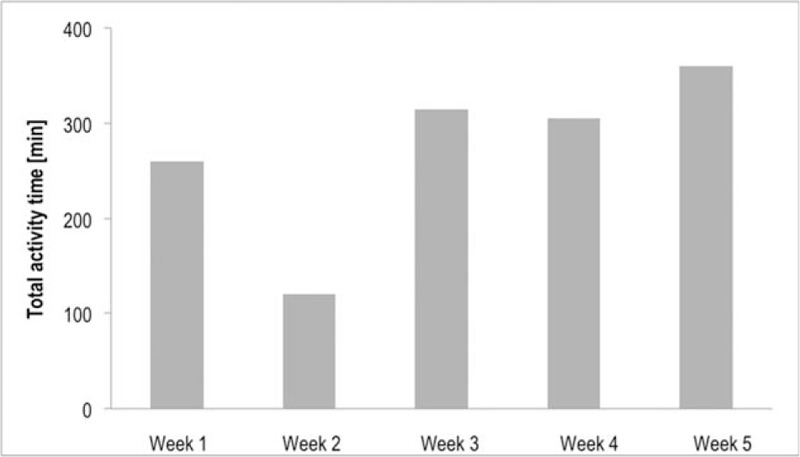
Self-reported weekly amounts of habitual physical activity, excluding prescribed HIIT sessions.

The self-reported weekly habitual physical activities of daily living are presented in Figure 2. No adverse events or new oncological indications were reported throughout the intervention and the 3-month follow-up.

#### 2.6.2 Physical functioning

**Figure 3:**
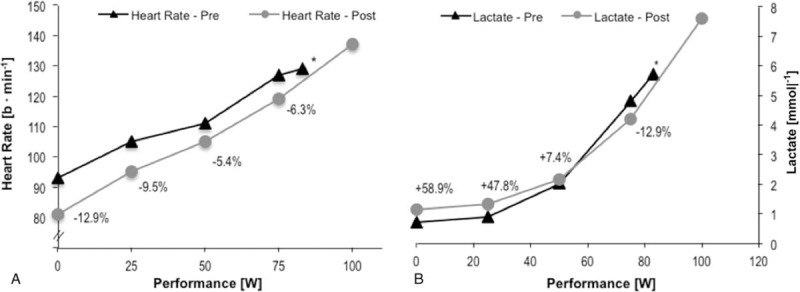
Heart rate (A) and blood lactate accumulation (B) during the cardiopulmonary exercise test before and after training; + indicates an increase from pre to post at the corresponding workload; - indicates a decrease from pre to post at the corresponding workload. ∗NOTE: the curves for pre are shorter due to a shorter time to exhaustion.

W_max_, VO_2peak_, and power at 4 mmol blood lactate concentration improved by 21% (83–100 W), 13% (23.0–26.1 mL  min^−1^ kg^−1^) and 7.3% (67.7–72.6 W), respectively. Simultaneously, heart rate corresponding to 4 mmoL blood lactate concentration decreased by 3.8% (122.3 to 117.7 b · min^−1^), whereas RPE remained unchanged (12.4 vs 12.8). The HR and blood lactate curves of the incremental exercise test are presented in Figure 3A and B.

Body weight, lean mass and BMI increased by 4.5%, 6.6% and 4.1% (49.1 to 51.3 kg; 33.5–35.7 kg; 17.9–18.6 kg/m^2^), respectively. Simultaneously the percentage of body fat was reduced by 1.4% (31.8–30.4%).

Lung function as assessed by FVC and FEV_1_ remained stable with changes below 5%. The Tiffeneau–Pinelli index was reduced by 6.6% but remained with 84.7% within the normal range.

The total number of white blood cells as well as the content of neutrophils and mixed cells was decreased by 41.7%, 48.6%, and 89%, respectively (Table 3).

**Table 3:**
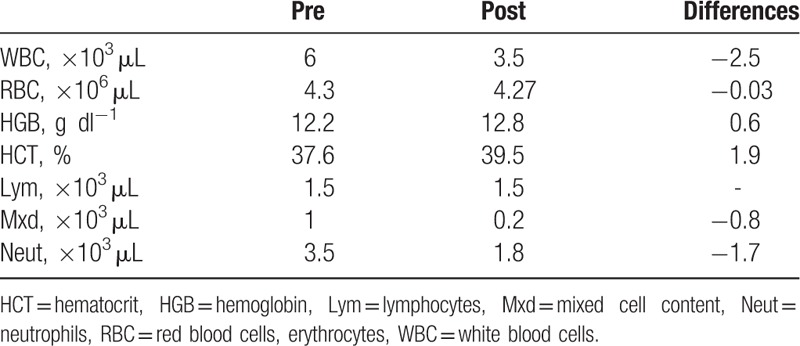
Basic blood counts at pre- and post- intervention.

Resting heart rate during supine and standing decreased by 14.5% and 10.5%, respectively. HF and LF during supine increased by 5-fold and 1.5-fold, respectively, while during standing HF increased 3-fold, and LF decreased by 6.5% (Table 4).

**Table 4:**
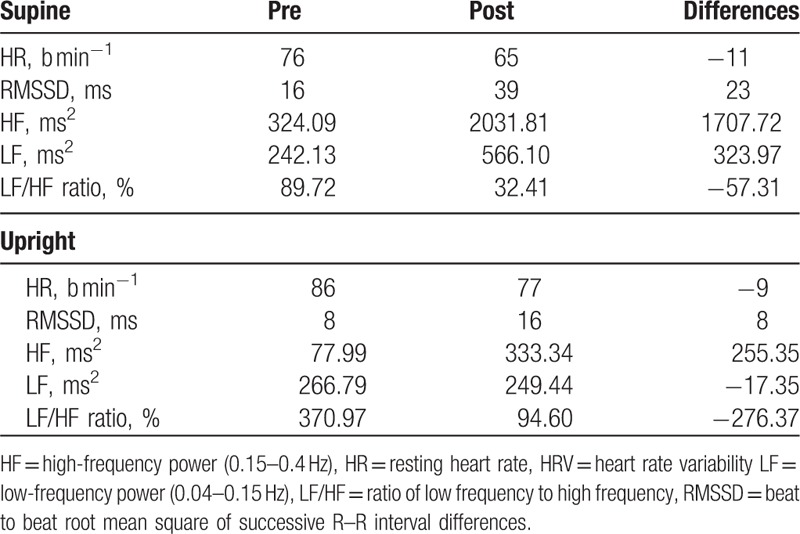
Measures of the orthostatic test and HRV, including the differences between pre- and post-intervention.

#### 2.6.3 Patient-related outcomes

**Figure 4:**
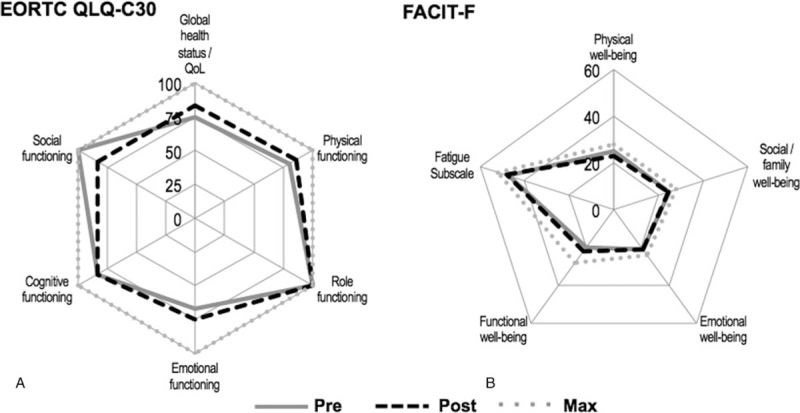
Patient-related outcomes on quality of life assessed by the EORTC QLQ-C30 functional scale (A) and perceived fatigue determined by the FACIT-F subscales (B) before and after the intervention in relation to the maximal values. NOTE: The FACIT-F questionnaire was assessed both before the first and last HIIT session. FACIT-F = functional assessment of chronic illness therapy-fatigue.

The development of the functioning subscales of the EORTC QLQ-C30 and the well-being subscales FACIT-F are shown in Figure 4A and B, respectively. In the EORTC QLQ-C30, physical functioning showed a positive development of 7.5%, while the physical well-being score of the FACIT-F showed an 8% reduction. Furthermore, in EORTC QLQ-C30 the social functioning score decreased by 17%, while the FACIT-F scores for social and family well-being slightly decreased by 2%. In addition, in the EORTC QLQ-C30 emotional functioning was improved by 11.9%, while the score for emotional well-being of the FACIT-F remained unchanged. All cancer-related symptom scales (fatigue, nausea and vomiting, pain, dyspnea, insomnia, appetite loss, constipation, diarrhea, and financial difficulties) of the EORTC QLQ-C30 remained unchanged. However, FACIT-F-assessed functional well-being and the score of the fatigue subscale improved about 10% and 2.1%, respectively. The global health status of the EORTC QLQ-C30 improved for 10.7% (Fig. 4A). The BPI-SF and PSQI showed no alteration. The FACT-Cog revealed a reduced score for perceived cognitive impairment by 9.8%, while the remaining 3 subscales (impact of perceived cognitive impairment, and perceived cognitive abilities and comments from others) remained unaltered.

## 3 Discussion

In this case report we showed that HIIT with an increased FiO_2_ was safe and feasible in a CRC patient concurrently undergoing chemotherapy. In fact, already 8 sessions of HIIT with concomitant hyperoxia improved physical functioning, as represented by improvements in cardiorespiratory fitness and power output as well as body composition, QoL and autonomous nervous function. To the best of our knowledge, this is the first case study focusing on the feasibility, safety and changes in physical functioning of a combined HIIT and hyperoxia intervention in a cancer patient undergoing chemotherapy.

The present case was conducted in a heavily deconditioned middle-aged and inactive woman, diagnosed with hepatic metastatic colon carcinoma and evidence of further cardiovascular risk factors (daily alcohol and nicotine abuses; I10.90; E78.2). The patient was treated with FOLFOX4, which is known to induce emesis, vomiting, fatigue and neuropathy.^[3]^ Nevertheless, we showed clinically relevant improvements in physical functioning, such as improved aerobic capacity, body composition, QoL and HRV even after 4 weeks of HIIT. Furthermore, no adverse events were reported, indicating that HIIT may be safely performed in CRC patients undergoing chemotherapy.

Our findings of improved physical performance were in line with a recent study by Devin et al. (2016), who reported similar improvements of HIIT for VO_2peak_ and W_max_ of 20.2% and 18.8% over 4 weeks in CRC cancer patients post-treatment.^[12]^ In addition, the observed changes in body composition and increased levels of habitual physical activity after the intervention were similar to our findings.^[12]^ Interestingly, the training volume and frequency were much higher in the study by Devin et al. as compared to our case and the adherence to the total training time differed tremendously with 99.7% vs only 51% in our study. This is somewhat surprising and future studies should investigate whether these differences may at least be partly associated with the increased FiO_2_, e.g. by implementing a normoxia control group.

The low training adherence of only 51% in the present case was not expected, particularly because hyperoxia is known for reducing subjective perceived exertion and enhancing physical performance.^[13]^ Current evidence suggests, that hyperoxia during exercise can counteract limited cerebral oxygenation and lead to an improved cellular diffusion of oxygen and oxygen-availability in the working muscles.^[21]^ However, even with an increased FiO_2_ during each exercise session, the intensity of 90% W_max_ appeared to be too high, which is indicated by the low overall training adherence. However, the overall training compliance in terms of training attendance was excellent, indicating a high motivation to perform strenuous exercise even concomitantly to the chemotherapy. Moreover, no adverse advents were reported throughout the intervention and during the 3-month follow-up.

Consistent with other studies investigating the effects of exercise in CRC patients, we also showed improvements in patient-related outcomes, such as QoL as well as emotional and physical functioning.^[22,23]^ Interestingly, the EORTC QLQ-C30 revealed no changes in cancer-related symptom scales, including fatigue, while the FACIT-F revealed a slight improvement in the fatigue subscale. However, it should be noted that the EORTC QLQ-C30 was collected pre- and post-intervention, whereas the FACIT-F was completed prior to the first and last HIIT session. Since both questionnaires are reviewing the previous 7 days, it is likely that the EORTC QLQ-C30 reflected the chronic responses of the intervention, while the FACIT-F rather captured the acute fatigue response to the training intervention. Another aspect aiding to explain the differences in the outcomes of the 2 questionnaires might be the relatively short period between chemotherapy cycle 5 and completion of the EORTC QLQ-C30, because drug administration was only 3 days prior to the post-intervention assessment.

FOLFOX4-induced neurotoxicity might not only alter patient-related outcomes like QoL and fatigue but may also lead to neuropathies, e.g. reflected in declined autonomous nervous function.^[2,24]^ In this case report we showed that a combination of HIIT and hyperoxia performed during chemotherapy actually improve autonomous nervous function by increasing the HRV components of HF and LF power as well as RMSSD during an orthostatic test. Recent studies showed that a higher HRV is associated with a lower level of carcinoembyonic antigens, a reduced cancer-related fatigue symptomatic, an improved QoL and a higher rate for overall survival in cancer patients.^[25,26]^ Therefore, the increased RMSSD and HF power might at least aid explaining the slight decrease of the FACIT-F fatigue subscale. However, the correlation of HF and LF power with symatho-vagal balance is still controversially discussed ^[27]^ and, thus, our results regarding the autonomous remodeling through chemo-toxicity remain preliminary.

Another well-known side-effect of FOLFOX is neutropenia.^[3]^ The reduction of 48.6% in neutrophils and 80% in the mixed cells content, mainly accounted for the observed decrease in white blood cells. Although an absolute neutrophil count of 1.8∗10^9^/L is not considered a clinical neutropenia, it might indicate the late onset of a chemotherapy-induced neutropenia. Interestingly, chemotherapy-induced neutropenia may be associated with an increased overall survival rate in CRC patients as shown in a recent meta-analysis.^[28]^ The underlying mechanisms for this correlation, however, are not fully understood, but might be related to a higher cancer stem cell death and may, thus, provide information about the level of drug exposure, dose density delivered and metabolic activity.^[28]^ However, not only the administration of medication might influence neutrophil count but also both high-intensity interval training and hyperoxia may alter neutrophil activation due to enhanced reactive oxygen species (ROS) production and accumulation.^[29,30]^ Increased ROS production, in turn, may initiate immigration of neutrophils into the lung tissue, which would also be reflected in a decreased WBC content.^[29]^ Thus, the impact of hyperoxia with combined intensive exercise loading under chemotherapy on physiological responses (e.g., ROS production, recruitment of inflammatory cells or tumor biology) needs further investigation.

The nature of a case study brings about a number of limitations, which should be considered when interpreting the present findings. First, the effects of both chemotherapy and exercise training are highly individual and the present training might not be feasible for all patients at any stage of treatment. Furthermore, it is difficult to distinguish whether the observed adaptations were solely induced by the exercise stimulus, the increased oxygen saturation or a combination of both. Last, the rather short intervention time may have not been sufficient to induce remarkable side-effects of the FOLFOX4 and, thus, it remains to be investigated whether similar beneficial effects of the exercise training would be observed when training and therapy span over a prolonged time of several months.

## 4 Conclusion

Despite the training having been well tolerated and safe in the present case, the combination of HIIT and hyperoxia also induced clinically relevant changes in physical functioning, such as physical fitness, body composition and autonomous nervous function. Improvements in physical functioning may contribute to better patient-related outcomes like an enhanced QoL and reduced cancer-related fatigue. To learn more about the underlying physiological mechanisms of an increased FiO_2_ during HIIT in cancer, further research, including measures of deep tissue oxygen saturation and delivery as well as a tumor marker readouts are needed. Thus, the findings of the present case should be further evaluated in randomized controlled trials including a control group with HIIT performed in normoxia, in order to identify the ideal combination of load, deload and duration of HIIT with altered FiO_2_ during chemotherapy.

## Acknowledgments

The authors would like to thank the patient for agreement and participation in this case study. We acknowledge the support of the German Research Foundation (DFG) and open access publishing fund of the University of Potsdam.

## Author contributions

**Conceptualization**: Wilhelm Bloch, Moritz Schumann.

**Data curation**: Nils Freitag, Pia Deborah Weber, Tanja Christiane Sanders, Wilhelm Bloch, Moritz Schumann.

**Investigation:** Nils Freitag, Tanja Christiane Sanders, Holger Schulz, Wilhelm Bloch, Moritz Schumann.

**Methodology:** Nils Freitag, Wilhelm Bloch, Moritz Schumann.

**Supervision:** Moritz Schumann.

**Visualization:** Nils Freitag.

**Writing – original draft:** Nils Freitag, Pia Deborah Weber, Tanja Christiane Sanders, Holger Schulz, Wilhelm Bloch, Moritz Schumann.

**Writing – review & editing:** Nils Freitag, Pia Deborah Weber, Tanja Christiane Sanders, Holger Schulz, Wilhelm Bloch, Moritz Schumann.
